# UK Guidelines for the Management of Bone Sarcomas

**DOI:** 10.1155/2010/317462

**Published:** 2010-12-29

**Authors:** Robert Grimer, Nick Athanasou, Craig Gerrand, Ian Judson, Ian Lewis, Bruce Morland, David Peake, Beatrice Seddon, Jeremy Whelan

**Affiliations:** ^1^Royal Orthopaedic Hospital, Birmingham B31 2AP, UK; ^2^Nuffield Orthopaedic Centre, Oxford OX3 7LD, UK; ^3^Freeman Hospital, Newcastle-upon-Tyne NE7 7DN, UK; ^4^The Royal Marsden, Sutton SM2 5PT, UK; ^5^Leeds Teaching Hospitals, LS9 7TF, UK; ^6^Birmingham Children's Hospital, B4 6NH, UK; ^7^Queen Elizabeth Hospital, Birmingham B15 2TH, UK; ^8^University College Hospital, London NW1 2BU, UK

## Abstract

These guidelines have been developed in order to provide an overview and a set of broad-based key recommendations for the management of patients with bone sarcomas in the UK. They have taken into consideration the most up-to-date scientific literature along with the recent recommendations by the European Society of Medical Oncology. The principles of the NICE guidance on both “improving outcomes for patients with sarcomas” and “improving outcomes with children and young people with cancer” have been incorporated. As care evolves, it is acknowledged that these guidelines will need updating. The key recommendations are that bone pain or a palpable mass should always lead to further investigation and patients with clinicoradiological findings suggestive of a primary bone tumour should be sent to a reference centre. Patients should then have their care managed at such a specialist centre by a fully accredited multidisciplinary team.

## 1. Introduction

### 1.1. Rationale and Objective of Guidelines

Bone sarcomas are an uncommon group of malignancies. It was recognised more than 20 years ago in England that management of bone sarcomas should be centralised; this led to the recognition of two supraregional centres by the National Specialist Commissioning Advisory Group (NSCAG) [now National Commissioning Group (NCG)] in 1986. Until now, however, there have been no clinical guidelines to document the standard of care for patients with these tumours. 

In the US, the National Comprehensive Cancer Network (NCCN) bone sarcoma guidelines [1] are highly regarded as are those developed by The European Society of Medical Oncology (ESMO) in conjunction with EUROBONET [2–4]. Using these documents as a framework, clinical management guidelines for patients with bone sarcoma in the UK were drawn up by consensus under the auspices of the British Sarcoma Group (BSG). Levels of evidence [I–V] and grades of recommendation [A–D] as used by the American Society of Clinical Oncology [5] have been published recently in the ESMO guidelines and are also referred to elsewhere [6, 7]. We have not included the levels of evidence in this paper.

These guidelines are not intended to be prescriptive, but aim to educate and improve the quality of care for patients with bone sarcomas by helping to identify and inform the key decisions involved in their management. Equally, this paper does not extend to rehabilitation, prosthetic services, and palliation.

### 1.2. Methods

The NCCN and ESMO guidelines together with the National Institute for Health and Clinical Excellence (NICE) Improving Outcomes Guidance for people with sarcoma [8] were used as the basis for discussion by the group, adding or omitting detail only where it was clearly agreed by consensus, in relation to UK specific issues. A recent literature review has been included to ensure that referencing is comprehensive.

### 1.3. Scope of Guidelines

The recommendations apply to all bone sarcomas arising in any skeletal location. 

These guidelines focus on clinical effectiveness, giving a picture of what treatments a specialist sarcoma multidisciplinary team (MDT) should have access to within the UK, subject to some flexibility to allow for evolving practice, but they do not employ the same detailed analysis of cost effectiveness as NICE. This material can be considered to represent a broad consensus in 2010; however, we acknowledge that it will require updating as treatment evolves.

## 2. Overview

### 2.1. Classification and Clinical Presentation

Primary bone tumours are rare, accounting for less than 1% of cancers in adults [9]. There are on average 427 new cases per year in England and Wales [8] Data from the Office of National Statistics and Welsh Cancer Intelligence and Surveillance Unit. They have a relatively high incidence in children and adolescents (accounting for approximately 5% of all childhood cancers in European Countries) [10], but can arise at any age. Given their rarity, nonspecialised clinicians, radiologists, and pathologists may find them difficult to recognise. To avoid diagnostic and management difficulties early referral to a specialist MDT prior to biopsy is important [11]. 

In adulthood, metastatic carcinomas and haemopoietic malignancies in bone considerably outnumber primary bone tumours. Where there is diagnostic uncertainty, it should be assumed that the patient has a primary bone sarcoma until proven otherwise [12]. 

During a working lifetime, a General Practitioner (GP) is unlikely to see a patient with a bone sarcoma. Delays in diagnosis are, therefore, common and earlier diagnosis would almost certainly lead to improved outcomes both in terms of survival and less extensive surgery.

The most common symptom of a primary bone tumour is pain which may be nonmechanical or night pain. The presence of bone pain at night should always be considered to be a “red flag” symptom leading to further investigation. The presence of bone swelling or a soft-tissue mass may occur later. The average duration of symptoms is 3 months although a history of 6 months or longer is not uncommon [13–15]. The presence of pain or a palpable mass arising from any bone should cause concern and lead to further investigation of which a plain X-ray is the first investigation of choice. The presence of any of the following on the X-ray is suggestive, but not diagnostic of a bone tumour and should also lead to further investigation:

- bone destruction,
- new bone formation,
- periosteal swelling,
- soft tissue swelling.

The histological classification of primary malignant bone tumours according to the World Health Organisation (WHO) is given in Table 1.

Although listed by the WHO as bone tumours, the management of haemopoetic tumours of bone is beyond the scope of these guidelines.

### 2.2. Epidemiology and Aetiology

Although there are a number of inherited and acquired factors associated with the development of primary bone tumours, it is not possible to identify a particular cause in the majority of patients [17, 18].

#### 2.2.1. Osteosarcoma

Osteosarcoma is the most frequent primary cancer of bone (incidence 0.2-0.3/100,000/year) [2], UK rates are 0.27/100,000 population in England [19]. There is an average of between 124–150 cases per year in England and Wales [8, 19]. The incidence is higher in adolescents (0.8–1.1/100,000/year at age 15–19), where it accounts for >10% of all solid cancers. The male-female ratio is 1.4 : 1 (UK data 1.1 : 1-NCIN) The majority arise in adolescence, but some are linked to other pathologies in the seventh and eighth decades of life [20]. 

Osteosarcoma usually arises in the metaphysis of an extremity long bone, most commonly around the knee [21, 22]. Some tumours (predominantly in adults) arise in the axial skeleton or craniofacial bones. Conventional osteosarcoma, a high-grade malignancy, accounts for the majority of osteosarcoma. Its most frequent subtypes are osteoblastic, chondroblastic, and fibroblastic. Other high-grade types are telangiectatic, small cell, and high-grade surface osteosarcoma. Low-grade central and parosteal osteosarcoma are low-grade malignancies, while periosteal osteosarcoma is an intermediate-grade chondroblastic osteosarcoma. Risk factors for the occurrence of osteosarcoma include previous radiation therapy, Paget's disease of bone, and germ line abnormalities such as the Li-Fraumeni syndrome, Werner syndrome, Rothmund-Thomson syndrome, and familial retinoblastoma [23, 24].

#### 2.2.2. Ewing Sarcoma

Ewing sarcoma (including primitive neuroectodermal tumour of bone) is the second most common primary malignant bone cancer in children and adolescents, but is also seen in adults. The median age at diagnosis is also around 15 years and there is a male predilection of 1.5 : 1 (UK data 1.2 : 1 NCIN). Ewing sarcoma is diagnosed in children aged 0–20 at an incidence of 0.3/100,000/year [UK all age standardised rates report 0.11-0.12/100,000 population in the UK (1979–2004)] with approximately 65–75 new cases per year in the UK [8, 19]. The most frequent sites of involvement are the long bones, pelvis, ribs, and vertebral column. It is less common in people of Chinese or Black African origin. All forms of Ewing sarcoma are high-grade tumours [3, 25].

#### 2.2.3. Chondrosarcoma

Chondrosarcoma is one of the most common bone sarcomas of adulthood, characterised by the production of tumour cartilage [26]. The age standardised incidence may be as high as 0.25/100,000 per year in males and 0.2/100,000 in females per year [UK age standardised rates report 0.19/100,000 population in the UK (NCIN)], with the most common age being between 30–60 years [27]. There are approximately 100–120 new cases per year in the UK [8]. 

The majority of primary chondrosarcomas are low rather than high grade [28]. Most chondrosarcomas are located in the long bones, but they also occur in flat bones such as pelvis, rib, and scapula. Chondrosarcoma can arise in preexisting benign lesions such as osteochondroma and enchondroma. In these circumstances, they are referred to as secondary peripheral chondrosarcomas and secondary chondrosarcomas, respectively.

The majority of chondrosarcomas are of the conventional subtype, but rarer subtypes include mesenchymal chondrosarcoma and clear cell chondrosarcoma. In rare circumstances, conventional chondrosarcomas can “dedifferentiate” into a very high-grade tumour with a dismal prognosis so-called dedifferentiated chondrosarcoma [26, 29, 30]. Most chondrosarcomas are solitary, but they can occur as multiple lesions in patients with osteochondromatosis (hereditary multiple exostoses) and enchondromatosis (Ollier's disease). The risk of malignancy in solitary enchondromas and osteochondromas is uncertain, but it is increased when there are multiple lesions.

#### 2.2.4. Spindle Cell Sarcomas of Bone

Spindle cell sarcomas of bone represent between 2% and 5% of primary bone malignancies and include a variety of diagnostic groups including fibrosarcoma, malignant fibrous histiocytoma (MFH), leiomyosarcoma, and undifferentiated sarcoma. These tumours arise in a similar age group to chondrosarcoma, but the skeletal distribution is more like that of osteosarcoma. They typically present with pain and have a high incidence of fracture at presentation. Radiological characteristics include a punched out or lytic appearance. An association with pre-existing disease/abnormality (Paget's disease or bone infarct) or history of previous irradiation has been reported. It is not unusual for a spindle cell sarcoma to be found to be either a dedifferentiated chondrosarcoma or an osteosarcoma after resection.

#### 2.2.5. Other Bone Sarcomas

These include such entities as adamantinoma and chordoma which both have specific clinical presentation and management [16]. Adamantinoma of bone typically arises in the anterior cortex of the diaphysis of the tibia. Chordomas are rare, typically arising in the sacrum or base of skull from notochord remnants. 

**Figure 1:**
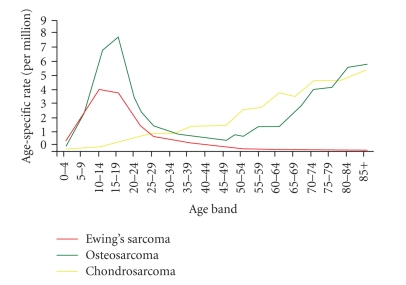
Age-specific incidence rates by morphology, England, 1979–2004.

Age-specific incidence rates by morphology are shown in Figure 1.

## 3. Diagnosis and Referral

### 3.1. Early Steps in Diagnosis

In children under the age of 5 years, primary malignant bone tumours are rare. Between the age of 5 and approximately 40, a primary malignant bone tumour is likely to be the most common diagnosis of a bone neoplasm in the absence of a past history of malignancy. Over 40, the incidence of metastases (usually from bronchus, breast, thyroid, kidney, or prostate) becomes increasingly common. At any age, the possibility of a benign lesion, infection, or haematological malignancy (plasma cell tumor or lymphoma) must be considered.

In all patients a full clinical history (duration/intensity of complaints, time points of pain during the day, prior benign and malignant lesions, family history, and previous radiotherapy) and examination (with specific attention to size/consistency of swelling, location and relation of swelling to bone, demarcation and mobility of swelling and palpation of regional, and local lymph nodes) should be performed, taking into account the most likely diagnoses listed above. In patients under the age of 40, investigations prior to referral should include X-ray of the affected bone and chest as well as simple blood tests [(full blood count (FBC), erythrocyte sedimentation rate (ESR), biochemical profile including alkaline phosphatase (ALP)]. Magnetic resonance imaging (MRI) can be done prior to or after referral [15].

In patients over the age of 40, where metastatic disease is more common, patients should be more extensively investigated to exclude a primary tumour at another location, ideally with computed tomography (CT) of the chest, abdomen and pelvis, isotope bone scan, and a myeloma screen prior to referral—provided these can be done quickly. If the lesion is solitary and no primary site identified, then the patient should be referred to one of the reference centres.

 All patients with clinicoradiological findings suggestive of a primary bone tumour should be sent to a bone tumour reference centre prior to biopsy. In England, there are five designated centres.

### 3.2. UK Reference Centres

- Royal National Orthopaedic Hospital: London, phone 020 8909 5584/5600, fax 020 8909 5709, http://www.londonsarcoma.org.

(ii) Royal Orthopaedic Hospital: Birmingham, phone 0121 685 4150, Fax 0121 685 4146.

(iii) Nuffield Orthopaedic Centre: Oxford, phone 01865 738061.

(iv) North of England Bone and Soft Tissue Tumour Service, Freeman Hospital:: Newcastle upon Tyne phone 0191 223 1496, fax 0191 233 1328, http://www.newcastlesarcoma.org.uk.
(v) Greater Manchester and Oswestry Sarcoma Service, Robert Jones and Agnes Hunt Hospital: Oswestry, phone 0845 838 3429, fax 0845 838 3428.

### 3.3. Management at Reference Centre

All patients with a suspected bone sarcoma should be referred for diagnosis and have their care managed at a reference centre by a fully accredited multi disciplinary team (MDT) [31, 32]. This is a core principle of the NICE Guidance “improving outcomes for patients with sarcomas” [8] and “improving outcomes with children and young people with cancer” [33]. This guidance also recommends the following.

- Networks should ensure that GPs are aware of and comply with the urgent referral criteria in the NICE “referral guidelines for suspected cancer” and that GPs and hospital doctors are aware of the diagnostic pathways.
- All patients with a confirmed diagnosis of bone sarcoma should have their care supervised by or in conjunction with a sarcoma MDT and be allocated a key worker. Children, teenagers, and young adults should also be discussed at the relevant children's or TYA (young adult) MDT.
  - Networks should ensure that they meet the needs of children and young people with cancer with sufficient specialist staff and care and facilities appropriate to the child or young persons age.
- A bone sarcoma MDT should meet minimum criteria for the number of patients treated in each year and adhere to the requirements for core membership of the relevant specialties.
  - All sarcoma MDTs should collect data on patients, tumours, treatment, and outcomes as agreed nationally.
- All patients with a provisional histological and/or radiological diagnosis of bone sarcoma should have their diagnosis reviewed by a specialist sarcoma pathologist and/or radiologist who are part of a sarcoma MDT.

(v) Patients should undergo definitive resection of their sarcoma by a surgeon who is a member of a sarcoma MDT or by a surgeon with tumour site-specific or age-appropriate skills, in consultation with the sarcoma MDT.

(vi) Chemotherapy and radiotherapy are important components of the treatment of some patients and should be carried out at designated centres by appropriate specialists as recommended by a sarcoma MDT.

(vii) Patients should be informed about relevant clinical trials and supported to enter them.

Furthermore, referral of patients to the reference centre for initial biopsy is also strongly recommended. This obviates the risk associated with improperly performed biopsies and increases the proportion of patients whose tumours can be studied with modern molecular techniques.

## 4. Investigation

### 4.1. Imaging

All patients should have plain X-rays in two planes at presentation. CT should only be used in cases where there is diagnostic uncertainty and to optimally visualise areas of microcalcification, periosteal bone formation, cortical destruction, or soft tissue involvement. CT can also be helpful for surgical planning in the pelvic area. When the diagnosis of malignancy is possible on radiographs, MRI of the whole bone is the next most appropriate imaging investigation and is the best modality for local staging [34].

General staging (where indicated) to assess the extent of distant disease should include chest radiography and/or CT. CT remains the diagnostic technique of choice for imaging the chest [35], (appreciating that small nodules are not specific for malignancy). Whole body bone scintigraphy will detect lesions elsewhere in the skeleton [3]. Whole body MRI and positron emission tomography (PET) are under evaluation both for staging and treatment response evaluation [36]. Additional appropriate imaging studies and biopsies should be taken of suspicious sites, as accurate staging of the disease has an impact on treatment and outcome. Guidance on the management of small suspicious lung nodules is available in trial protocols [37, 38]. 

In the case of chondrosarcoma, a contrast enhanced MRI can reveal high-grade areas, which is useful to guide the site of biopsy.

### 4.2. Staging

There are two common staging systems in use, the Enneking [39] and the TNM system (American Joint Committee on Cancer-AJCC/International Union against cancer-UICC) [40].

The Enneking system is based on tumour grade (I = low and II = high grade) and extent in relation to the anatomical compartments of the limb. Where the bone cortex is intact, the tumour is deemed to be intracompartmental. Stage III tumours can be any grade with metastases. This is summarized in Table 2.

The TNM (AJCC/UICC) system is based on tumour grade, size, and the presence of metastases (Table 3).

### 4.3. Laboratory Tests

No specific laboratory tests for diagnosis of bone sarcoma are available. However, some may be of prognostic value; examples include ESR, alkaline phosphatase (ALP) and lactate dehydrogenase (LDH) [41, 42].

### 4.4. Other Baseline Assessments

Chemotherapy treatment can result in renal, cardiac, and auditory dysfunction [43], and patients undergoing this treatment must have baseline renal function testing and assessment of cardiac function as well as an audiogram (in case of treatment with cisplatin). Sperm storage is recommended for male patients of reproductive age. For female patients, a fertility physician should be consulted to discuss options with the patient.

### 4.5. Biopsy

The definitive test remains biopsy. The biopsy of a suspected primary malignant bone tumour should be carried out at the reference centre by the surgical team who are to carry out the definitive tumour resection. Inappropriate techniques can irrevocably compromise the chance of limb salvage or even cure. The principles of biopsy [11] are the following:

- Biopsy should only be done after local imaging of the affected bone to allow planning of the most representative area to biopsy.
- There should be minimal contamination of normal tissues.
- In many situations, core needle biopsy will be adequate, often controlled by ultrasound, X-ray or CT.
- Samples should always be taken for micro biological culture as well as histology and cytogenetic studies.
- In the reference centre, samples should ideally be snap frozen for storage in a tumour bank for future translational research studies (patient consent should be obtained).
- Samples must be interpreted by an experienced pathologist.
- The request form should contain sufficient detail for a pathologist to make a diagnosis, including the site of the tumour, the patient's age, and the radiological differential diagnosis.

CT guided biopsies [44, 45] are appropriate for deeper locations (e.g., pelvis) or to target a particular area of concern (e.g., a possibly dedifferentiated area in a chondrosarcoma). In cases of uncertainty that the tissue is representative, frozen section may be considered in case more material is required. In aggressive and malignant tumours of bone, the biopsy track should be considered to be contaminated with tumour and must be removed together with the resection specimen at definitive surgery to avoid local recurrence; this includes possible channels through which drains have been placed. Biopsy tracks should be clearly marked by means of a small incision to ensure that the location can be recognised at the definitive procedure. 

Biopsy of other indeterminate lesions should always be considered if it will affect patient management (e.g., entry into a trial or local control decisions).

In cases of spinal column involvement, laminectomy or decompression should be avoided unless necessary to relieve spinal cord compression, and only after consultation with members of the bone sarcoma MDT.

### 4.6. Pathology

The pathologist reporting on both the diagnosis and resection of bone sarcomas should be an accredited bone tumour pathologist and part of a bone sarcoma MDT. The report should comply with guidance from the Royal College of Pathologists [46].

The biopsy report should include a description of the specimen received and the microscopic findings including the histological diagnosis. Histopathology reports of resection specimens should include a gross specimen description which records the location and size (measured in three dimensions in mm) of the tumour in the resected bone. The histological features of the tumour should be described and the tumour type (and subtype) classified according to the latest WHO criteria [16]. The pathology report should note the extent of local tumour spread, including involvement of specific anatomical compartments. Whether the resection margins are clear or involved should be noted and the distance (in mm) of infiltrating tumour from the nearest resection margin and the nature of tissue at this resection margin specified. Results of relevant ancillary investigations (e.g. immunohistochemistry or molecular genetics) should be recorded and the tumour should be coded using the Systematized Nomenclature of Medicine—Clinical Terms (SNOMED-CT) codes [47].

The extent of tumor necrosis in response to any preoperative therapy should be assessed as being greater or less than 90% necrosis in broad terms although much greater specificity can be obtained.

### 4.7. Molecular Genetics and Pathology

Tumour banks are instrumental for diagnostic and translational research in the molecular pathology of cancer; therefore, informed consent for tumour banking should be sought that allows for later analysis and research according to local practice. In specialist centres, storage of fresh frozen tissue should be undertaken in every case where consent has been given.

Although most Ewing sarcomas can be recognised morphologically and by immunohistochemistry for the surface glycoprotein CD99, Ewing sarcoma translocation detection is mandatory when the clinicopathological presentation is unusual or the histological diagnosis is doubtful. A reference laboratory for Ewing sarcoma diagnosis should have both interphase fluorescence *in situ* hybridisation (FISH) and reverse transcription-polymerase chain reaction (RT-PCR) technology available [48]. The laboratory should be a participant in an external quality assurance programme.

### 4.8. Confirmation of Diagnosis

In every case, the diagnosis must be confirmed by reference to clinical findings, laboratory investigation and, in particular, radiological imaging at a sarcoma MDT. Ideally, all patients with suspected bone tumours should be discussed at the MDT meeting with the surgeon, the radiologist who has interpreted the imaging and the pathologist who has reviewed the biopsy material and an oncologist [8]; this will minimise the risk of errors in diagnosis and management.

## 5. Prognostic Factors

### 5.1. Osteosarcoma

Estimates of patients with newly diagnosed osteosarcoma with metastases, mainly located in the lung [14, 49, 50], vary from 12.4%–34.4% rising to 40% of all cases over time [51]. Microscopic, subclinical metastatic disease is present in the majority of patients at diagnosis. Adverse prognostic or predictive factors include detectable primary metastases as well as axial or proximal extremity tumour site, large tumour volume, elevated serum alkaline phosphatase or LDH, older age, and poor histological response to preoperative chemotherapy/level of chemotherapy induced necrosis [41, 52].

### 5.2. Ewing Sarcoma

Around 26% of patients are diagnosed with metastatic disease (10% lung, 10% bones/bone marrow, 6% combinations, or others) [25]. Bone metastases confer a poorer outcome than lung/pleura metastases (<21% compared with 55% 5-year relapse free survival) [53]. Other known prognostic factors are tumour site/axial location or volume, raised serum LDH levels and older age (>15 years). A poor histological response to preoperative chemotherapy and incomplete or no surgery for local therapy are further adverse prognostic factors [42, 52, 54, 55].

### 5.3. Chondrosarcoma

All chondrosarcomas have a significant risk of local recurrence, but metastases rarely arise in patients with grade I tumours. Grade II and III chondrosarcomas are often grouped together even though there is a wide spectrum of outcome—grade and stage are independent prognostic factors [56] along with histology [27]. Clear cell chondrosarcoma behaves like a low-grade chondrosarcoma, whilst mesenchymal chondrosarcoma should be considered a high-grade bone sarcoma. Dedifferentiated chondrosarcomas are high-grade aggressive sarcomas and frequently metastasise to lungs and other bones [26, 30].

### 5.4. Spindle Cell Sarcomas of Bone

Prognosis is similar to patients with osteosarcoma of the same age and is related to the same prognostic factors.

## 6. Management Overview

Bone sarcoma is a potentially curable disease with surgery and chemotherapy being the mainstays of treatment-notably preoperative “neoadjuvant” systemic combination chemotherapy, local surgery, and postoperative “adjuvant” chemotherapy [43]. One of the main goals is to decrease the incidence of a subsequent distant relapse [57]. Despite a lack of new treatments over time, outcomes have improved, due to use of more aggressive multimodal treatments and utilisation of surgery for sites previously considered inoperable [58]. 

Survival for patients with bone tumours has improved substantially over the last 30 years in the UK. A national study looking at the survival of patients aged 15 years or older with bone cancer of all types found that 5-year relative survival rates increased from 29% in 1971–1975 to 51% in1986–1990 [59]. Patterns of survival for patients under 40 in the UK showed an increase from 42% to 53% in the period from the early ‘80s to the early ‘90s for osteosarcoma and 31% to 51% for Ewing sarcoma [60]. The EUROCARE data for children aged 0–14 years showed an increase from 52% to 60% for osteosarcoma, and 50% to 60% for Ewing sarcoma during the latter 4 years of a survey performed between 1978 and 1989 when compared to the entire 11 year period [10]. Data from northern England and the West Midlands for the period 1981–2002 showed an improvement for Ewing sarcoma but not for osteosarcoma for a number of reasons including potential delays in diagnosis, accrual to trials, adherence to therapy and lack of improvement in treatment strategies [17].

### 6.1. Systemic Chemotherapy

As malignant primary bone tumours are rare and management is complex, the accepted standard is treatment in reference centres or within reference networks able to provide access to the full spectrum of care in predetermined partnerships with such centres [2, 8]. 

Therapy is usually given within the framework of prospective, often collaborative, clinical studies, or established treatment protocols. In cases of high-grade osteosarcoma, Ewing sarcoma or spindle cell sarcoma (neo) adjuvant therapy is indicated, preferably within the framework of international clinical trials.

### 6.2. Surgery

Surgery of the primary tumour should be performed only after adequate preoperative staging, striving to obtain adequate surgical margins. A wide enbloc resection of the affected part of the bone and soft tissue should be performed. Close surgical margins may be identified with (MRI-inert) haemoclips.

Decisions about the optimal surgical procedure (i.e., limb salvage or amputation) should be made as part of an MDT discussion, taking into account tumour and treatment factors (e.g., extent of tumour and response to neoadjuvant therapy). The type of surgical reconstruction will depend on patient and surgeon choice and experience following open discussion of the risks and benefits of different options. Surgical excision of local recurrence or metastatic disease should usually be considered following decisions being made by the MDT.

#### 6.2.1. Requirements for the Surgical Report

The surgeon should describe the surgical procedure carried out and indicate the tissues resected. The planned surgical margin should be identified along with any areas of concern. The type of reconstruction should be described and the postoperative care to be used. The use of prophylactic antibiotics and anticoagulants should be clearly stated. The specimen should be orientated to allow the pathologist to adequately describe the anatomical location of close surgical margins.

### 6.3. Radiotherapy

The role of radiotherapy in osteosarcoma and chondrosarcoma is limited, as these tumours are recognised as being relatively radioresistant. Thus, radiotherapy is only used as definitive treatment of a primary tumour if there is no surgical option. It can be usefully used to achieve local tumour control at least in the short to mid-term although it is unlikely to achieve long term tumour control. However, there is limited evidence that osteosarcoma can sometimes be controlled by radiotherapy alone if there has been a good subjective response to chemotherapy [61]. If radiotherapy is to be used as definitive local treatment, a radical dose of radiotherapy is required. Standard conformal radiotherapy may not be able to achieve an adequate radiotherapy dose to the tumour, in which case techniques such as IMRT (intensity modulated radiotherapy) may allow delivery of a higher radiotherapy dose [62]. In addition, insertion of pelvic spacer devices can enable displacement of bowel away from pelvic tumours, which can also facilitate delivery of a higher dose. Heavy particle therapy with protons or carbon ions, often in combination with photons, are being used increasingly in the treatment of primary bone sarcomas not amenable to surgery [63–65]. Excellent outcomes have been reported for skull base chondrosarcomas with proton beam radiotherapy achieving approximately 70%–90% local control rates when combined with surgery [66], and the reported early results in bone sarcomas are encouraging. However, this approach is still relatively new and is considered at present as exploratory. At present, there is no proton facility in the United Kingdom, but potentially suitable cases can be submitted to the UK Proton Panel for consideration for approval for funding for treatment at a facility overseas. 

Radiotherapy may be used postoperatively in osteosarcoma and chondrosarcoma in selected cases, if there are concerns regarding (usually soft tissue) resections margins, or possible soft tissue contamination, and if further surgery is not possible [63]. 

In contrast with osteosarcoma and chondrosarcoma, Ewing Sarcoma is undoubtedly a radiation-sensitive tumour, and as such, radiotherapy can be usefully utilised as part of management. It may be used as definitive local therapy if surgery is not possible, with curative intent. It may also be used in combination with surgery, for patients who have had a poor histological response to chemotherapy, or when there are concerns regarding surgical resection margins [67, 68]. There is also a potential role for whole lung radiotherapy (WLRT) for patients with lung metastases [69, 70], and indeed, WLRT is included as part of treatment for patients with lung metastases in the current Euro-Ewing-99 protocol. 

Radiotherapy can be used for all bone sarcoma types for palliation of metastatic disease.

### 6.4. Prevention and Management of Pathological Fracture

Where there is an existing pathological fracture in a possibly malignant primary bone tumour, adequate imaging should be performed including MRI followed by biopsy. In cases of fracture, internal fixation is contraindicated as it disseminates tumour further into both bone and soft tissues and increases the risk of local recurrence. External splintage is recommended, along with appropriate pain control. In patients with weakened bone apparent at presentation, there may also be a strong case for immobilising the part after biopsy.

Neoadjuvant chemotherapy should be used in the expectation that a good response will allow the fracture haematoma to contract and allow subsequent resection of the tumour and the involved soft tissues. In patients with a poor response to chemotherapy, or tumours unlikely to respond to chemotherapy then early surgery obtaining wide margins should be considered, which may require amputation. Postoperative radiotherapy may be considered to try to decrease the risk of local recurrence [71].

## 7. Specific Treatment

### 7.1. Osteosarcoma

#### 7.1.1. Localised Disease

Curative treatment for high-grade osteosarcoma consists of surgery and chemotherapy [2, 43]. Compared with surgery alone, multimodal treatment of high-grade osteosarcoma increases survival probabilities from only 10%–20% to around 60% [72].

The goal of surgery is to safely remove the tumour and yet preserve as much function as possible. Most patients should be considered candidates for limb salvage. Wide surgical margins should be planned accepting that an apparent good response of the tumour to chemotherapy may allow a closer margin of excision to be considered safe. 

Doxorubicin, cisplatin, high-dose methotrexate, and ifosfamide have demonstrated antitumour activity in osteosarcoma [31, 72–75] and are usually administered in protocols involving 3 or 4 drug combinations. These drugs should be administered with adequate supportive care by experienced paediatric oncologists or medical oncologists in reference institutions with appropriate infrastructure with a MDT approach [43]. A variety of pre- and postoperative combinations are used in common practice and in clinical trials, and the ideal combination regimen and the optimal treatment duration are yet to be defined or confirmed [2, 76]. 

Most current protocols include a period of preoperative chemotherapy. This has not been proven to add survival benefit over postoperative chemotherapy alone, although there are clear practical advantages [31, 77]. Treatment is commonly given over periods of 6–9 months. The extent of histological response to pre operative chemotherapy however offers important prognostic information [78], but altering postoperative chemotherapy on the basis of response is not recommended outside of ongoing trials [38], and it is not accepted as a reliable surrogate endpoint for overall outcome [43, 72]. The use of haematopoietic growth factors to increase dose intensity has not consistently resulted in improved survival of osteosarcoma patients [72] but may limit morbidity associated with myelosuppression. 

The immune modulator liposomal muramyl tripeptide (mifamurtide) added to postoperative chemotherapy demonstrated a statistically significant advantage in overall survival and a trend in event-free survival in a large randomised trial [79] and has been approved in Europe for patients under 30 with completely resected localised osteosarcoma.

Whenever possible, patients with osteosarcoma should receive chemotherapy in the context of prospective trials, which is regarded as standard of care. Chemotherapy is also recommended for older patients with osteosarcoma using adapted protocols [20].

Low-grade central and parosteal osteosarcoma are variants with lower malignant potential, which are treated by surgery only. Careful analysis of the resected tumour may show areas of high-grade change in which case the patient should be treated as for a conventional osteosarcoma. The exact role of chemotherapy has not been defined for periosteal and jaw osteosarcoma, but experience has shown that chemotherapy can be given as standard. Jaw and other craniofacial osteosarcomas present specific problems for management, especially to achieve local control and should always be referred to a reference centre and discussed at a sarcoma MDT before surgical intervention.

#### 7.1.2. Metastatic and Recurrent Disease

Patients presenting with metastatic osteosarcoma are a heterogeneous group and may be treated using the same regimens used for nonmetastatic osteosarcomas provided that surgical resection of all sites of disease is deemed feasible [80]. Approximately 30% of all patients with primary metastatic osteosarcoma and >40% of those who achieve a complete surgical remission become long-term survivors [2].

The management of recurrent osteosarcoma needs to take into account the timing of recurrence, the site of recurrence, and the number of metastases. Treatment for recurrent osteosarcoma is primarily surgical, be it local recurrence or metastatic. Prognosis is poor, with long-term postrelapse survival of less than a third [2].

Pulmonary metastasectomy can play a major role but only if complete removal of all metastases can be achieved [51], as the disease is otherwise almost universally fatal. More than a third of patients with a second surgical remission survive for >5 years, even patients with multiple recurrences may be cured as long as recurrences are resectable, and repeated thoracotomies are often warranted [81].

The role of second-line chemotherapy for recurrent osteosarcoma is less well defined than that of surgery, and there is no accepted standard regimen [2, 81]. The choice of agents may take into account the prior disease-free interval and often includes ifosfamide ± etoposide, or possibly docetaxel/gemcitabine. The use of second-line chemotherapy has been shown to correlate with limited prolongation of survival in patients with inoperable metastatic recurrences, and a positive correlation in operable disease was observed in one series [82–84]. Radiotherapy to inoperable sites may be indicated for palliation.

### 7.2. Ewing Sarcoma

#### 7.2.1. Localised Disease

With surgery or radiotherapy alone, 5-year survival is <10%. With treatment in current multimodality trials including chemotherapy, 5-year survival is *∼*60%–70% in localised and *∼*20%–40% in metastatic disease [3].

All current trials employ three to six cycles of initial chemotherapy after biopsy, followed by local therapy and another six to ten cycles of chemotherapy usually given at 2 or 3 week intervals based on current agreed national or international protocols. Treatment duration is thus 10–12 months. Agents considered most active include doxorubicin, cyclophosphamide, ifosfamide, vincristine, dactinomycin, and etoposide [25, 37, 52, 54, 68, 69, 85–90]. Virtually all active protocols are based on four to six drug combinations of these agents. Chemotherapy intensity is positively associated with outcome. High-dose chemotherapy with blood stem cell transplantation is still investigational. 

Despite lively debate, complete surgery, where feasible, is regarded as the best modality of local control given the higher risk of local recurrence when radiotherapy is used as sole treatment for the primary tumour. Radiotherapy alone should be considered if complete surgery is impossible or if it will be very disabling. Individual decisions about local therapy are frequently complex and should only be made by an experienced reference centre MDT in conjunction with the parents and family.

Postoperative radiotherapy should be given in cases of inadequate surgical margins and discussed where histological response in the surgical specimen was poor (i.e., >10% viable tumour cells). Tolerability of therapies in adults needs to be taken into account when transferring treatment protocols conceived for children and adults ≤40 years.

#### 7.2.2. Metastatic and Recurrent Disease

Patients with metastases at diagnosis are treated similarly to patients with localised disease but have a worse prognosis. Several nonrandomised trials have assessed the value of more intensive, time compressed or high-dose chemotherapy approaches, followed by autologous stem cell rescue, but evidence of benefit, resulting from trials, is pending [70, 91, 92]. In patients with lung metastases, whole lung irradiation may confer a survival advantage but firm data are lacking and a systematic review failed to confirm a survival advantage [93]. The role of surgical resection of residual metastases is less well defined. Patients with bone or bone marrow metastases and patients with recurrent disease still fare poorly, with 5-year survival rates of ~20% [3, 94].

Prognostic factors relate to site of and time to relapse: patients relapsing later than 2 years from initial diagnosis and with disease not involving bone marrow or multiple bones have a better outcome [95, 96]. Doxorubicin therapy is usually no longer feasible due to previously achieved cumulative doses. Chemotherapy regimens in relapse situations are not standardised and are currently often based on alkylating agents (cyclophosphamide, high-dose ifosfamide) in combination with topoisomerase inhibitors (etoposide, topotecan) or irinotecan with temozolomide. [85, 90].

Radiotherapy may be helpful to palliate local symptoms.

### 7.3. Chondrosarcoma

Assessing the grade of chondrosarcomas is difficult and variations in opinions, even between experts are common [28]. Low-grade cartilage tumours are unlikely to metastasise but may recur locally. Biopsy confirmed low grade central chondrosarcomas in the long bones of the limbs can be managed by curettage with or without adjuvant (e.g., phenol, cement, and cryotherapy) with a high chance of success. Low-grade peripheral chondrosarcomas (arising from osteochondromas) should be surgically excised, aiming to excise the tumour with a covering of normal tissue over it. Higher-grade chondrosarcomas (including clear cell chondrosarcoma), and all chondrosarcomas of the pelvis or axial skeleton should be surgically excised with wide margins [26, 29]. 

Recent evidence suggests that mesenchymal chondrosarcoma may be responsive to chemotherapy and may be considered for adjuvant or neoadjuvant therapy [97, 98]. There remains uncertainty about chemotherapy sensitivity of de-differentiated chondrosarcoma but it is often treated like osteosarcoma, with poorer outcome [30]. There is a very high risk of local recurrence following excision of dedifferentiated chondrosarcoma, particularly in the presence of a pathological fracture. If wide margins cannot be reliably achieved with limb salvage then amputation may be considered but the metastases remain a problem.

### 7.4. Spindle Cell Sarcomas of Bone

Treatment strategies mimic those of osteosarcoma, with age-adjusted chemotherapy and complete enbloc resection including any soft-tissue component.

### 7.5. Other Bone Sarcomas

Although conventional therapy for chordomas has in the past been complete surgical resection [99], there are encouraging results from high-dose radiotherapy using proton beams or carbon ion facilities [100, 101]. Assessment in a specialist centre with expertise in managing these tumours is essential to define the role of surgery and/or radiotherapy. Metastases are rare but local recurrence common. There is evidence of some effectiveness of molecular targeted agents [102]. 

Most adamantinomas are low-grade but will recur if not completely resected. Higher-grade areas either in the primary tumour or in the recurrence may require systemic therapy.

## 8. Treatment Evaluation

Imaging whilst the patient is on chemotherapy is limited to intermittent assessment of the primary and metastatic sites of bone disease by clinical means (pain and clinical measurement) conventional radiographs, and assessment of the lungs by CT [34].

### 8.1. Osteosarcoma

Change in the size and ossification of the tumour is not a reliable guide to tumour response to neoadjuvant chemotherapy. Assessment of MRI detected peritumoural oedema is helpful: its disappearance is a sign of a good treatment response [103]. Dynamic MRI is reliable, but requires sequential scans to evaluate change in tumour vascularity [104]. Assessment of response is usually only apparent after several cycles of chemotherapy.

### 8.2. Ewing Sarcoma

Change in the size of the soft tissue mass is easily evaluated on MRI and is a reliable indicator of tumour response [105]. Dynamic MRI is not as reliable as in osteosarcoma, as remaining small tumour foci may not be detected. Sequential fluorodeoxyglucose PET (FDG PET) evaluation may be of additional value [34]. 

Progressive disease whilst on chemotherapy may mandate changes in treatment or earlier primary local control measures. An increase in the size of a tumour may, however, be due to necrosis rather than tumour progression.

## 9. Followup

Followup is designed to detect either local recurrence or metastatic disease at a time when early treatment is still possible and might be effective [34]. Followup of high grade tumours should include both a physical examination of the tumour site and assessment of the function and possible complications of any reconstruction. Local and chest imaging should be the norm. Evidence of optimum frequency of followup and the best imaging investigations is still lacking [34].

For high-grade tumors, current protocols recommend follow up intervals of 2–4 months for the first 3 years after completion of therapy, every 6 months for year 4 and 5 and thereafter annually [2, 3]. 

For low-grade bone sarcomas, the frequency of followup visits may be reduced to 4–6 monthly for 2 years and then annually. Late metastases as well as local recurrences and functional deficits may occur >10 years after diagnosis in all tumours, and there is no universally accepted stopping point for tumour surveillance [56].

It is important to evaluate the long-term toxicity effect of chemotherapy and radiotherapy as well as immediate chemotherapy related complications [43]. Monitoring for late effects should be undertaken, depending on the chemotherapy protocol and radiation used and in conjunction with late effect services when available [54, 106, 107].

Secondary cancers may arise in survivors of bone sarcomas, either related to or independent of irradiation. Secondary leukaemia, particularly acute myeloid leukaemia, may rarely be observed following chemotherapy as early as 2–5 years after treatment [108, 109].

## 10. Key Recommendations

- The most common symptom of a primary bone tumour is pain which may be non mechanical or night pain. The presence of bone pain at night should always be considered to be a “red flag” symptom leading to further investigation. The presence of pain or a palpable mass arising from any bone should cause concern and lead to further investigation of which a plain X-ray is the first investigation of choice.
- The presence of radiological features including bone destruction, new bone formation, periosteal swelling, and soft-tissue swelling are suggestive, but not diagnostic of a bone tumour and should also lead to further investigation.
- All bone tumour patients with clinicoradiological findings suggestive of a primary bone tumour should be sent to a reference centre.
- The definitive diagnostic test is a biopsy. The biopsy of a suspected primary malignant bone tumour should be carried out at the reference centre by the surgical team who are to carry out the definitive tumour resection.
- The pathologist reporting on both the diagnosis and resection of bone sarcomas should be an accredited bone tumour pathologist and part of a bone sarcoma multidisciplinary team (MDT). The report should comply with guidance from the Royal College of Pathologists.
- In every case, the diagnosis must be confirmed by reference to clinical findings, laboratory investigation and, in particular, radiological imaging at a bone sarcoma MDT, this will minimise the risk of errors in diagnosis and management. All patients should have tissue stored for subsequent investigation with appropriate consent.
- Patients should have their care managed at a reference centre by a fully accredited MDT. The MDT should make the decision about chemotherapy but may delegate the responsibility to another centre. All surgery should be carried out at a specialist bone sarcoma centre.
- Curative treatment for high-grade osteosarcoma consists of surgery and chemotherapy. All patients should be considered for inclusion into national or international clinical trials.
- Treatment for recurrent osteosarcoma is primarily surgical, be it local recurrence or metastatic. The role of second-line chemotherapy for recurrent osteosarcoma is less well defined than that of surgery and there is no accepted standard regimen.
- For Ewing sarcoma, systemic treatment with chemotherapy is standard. Where possible, surgery is preferred for local control over radiotherapy alone.
- Management of chondrosarcoma is surgical excision with wide margins for all but low-grade central limb chondrosarcoma where curettage may be adequate. There are no data to support the routine use of chemotherapy.
- Standard follow up for all sarcoma cases is currently chest X-ray and clinical review. The role of regular-cross sectional imaging remains uncertain.

## Figures and Tables

**Table 1:** 2002 WHO classification of malignant bone tumours [16].

Osteogenic tumours	﻿Osteosarcoma	9180/3
	﻿Conventional	9180/3
	﻿Chondroblastic	9181/3
	﻿Fibroblastic	9182/3
	﻿Osteoblastic	9180/3
	﻿Telangiectatic	9183/3
	﻿Small cell	9185/3
	﻿Low-grade central	9187/3
	﻿Secondary	9180/3
	﻿Parosteal	9192/3
	﻿Periosteal	9193/3
	﻿High grade surface	9194/3

Ewing sarcoma/primitive neuroectodermal tumour	﻿Ewing sarcoma	9260/3

Cartilage	﻿Chondrosarcoma	9220/3
	﻿Central, primary, and secondary	9220/3
	﻿Peripheral	9221/3
	﻿Differentiated	9243/3
	﻿Mesenchymal	9240/3
	﻿Clear cell	9242/3

Fibrogenic tumours	Fibrosarcoma	8810/3
﻿Fibrohistiocytic tumours		
Haemopoietic tumours	Plasma cell myeloma	9732/3
	Malignant lymphoma, NOS	9590/3
Giant cell tumour	Malignancy in giant cell tumour	9250/3
Notochordal tumours	Chordoma	9370/3
Vascular tumours	﻿Angiosarcoma	9120/3
Smooth muscle tumours	Leiomyosarcoma	8890/3
Lipogenic tumours	﻿Liposarcoma	﻿8850/3
Miscellaneous tumours	Adamantinoma	9261/3

**Table 2:** Enneking staging.

Stage	Grade	Tumour	Metastasis
IA	G1 low grade	T1 cortex intact (intracompartmental)	M0
IB	G1 low grade	T2 cortex breached with soft tissue extension	M0
IIA	G2 high grade	T1 cortex intact	M0
IIB	G2 high grade	﻿T2 cortex breached with soft tissue extension	M0
IIIA	G1 or G2	T1	M1
IIIB	G1 or G2	T2	M1

**Table 3:** AJCC/UICC staging.

Stage	Tumour (T)	Node (N)	Metastasis (M)	Grade (G)
Stage IA	T1 (tumour 8 cm or less)	No	M0	G1, 2 low grade
Stage IB	T2 (tumour more than 8 cm)	No	M0	G1, 2 low grade
Stage IIA	T1	No	﻿M0	﻿G3, 4 high grade
Stage IIB	T2	No	M0	G3, 4 high grade
Stage III	T3 (discontinuous tumours in primary site)	No	﻿M0	﻿Any G
Stage IVA	Any T	No	M1a (lung)	Any G
Stage IVB	﻿Any T	﻿N1 (regional lymph nodes)	﻿Any M	﻿Any G
	Any T	Any N	M1b (other sites)	Any G
